# Activity Patterns in Relation to Dynamic Functional Network States: A Longitudinal Feasibility Study of Brain–Behavior Associations in Young Adults

**DOI:** 10.3390/brainsci16030327

**Published:** 2026-03-19

**Authors:** Najme Soleimani, Maria Misiura, Ali Maan, Sir-Lord Wiafe, Jennalyn Burnette, Asia Hemphill, Vonetta M. Dotson, Rebecca Ellis, Tricia Z. King, Erin B. Tone, Vince D. Calhoun

**Affiliations:** 1 The Tri-Institutional Center for Translational Research in Neuroimaging and Data Science (TReNDS), Georgia State University, Georgia Institute of Technology and Emory University, Atlanta, GA 30303, USA; nsoleimani1@gsu.edu (N.S.);; 2 Department of Psychology, Georgia State University, Atlanta, GA 30302, USA; 3 Department of Neurology, Brigham & Women’s Hospital, Boston, MA 02115, USA; 4 Department of Neurology, Harvard Medical School, Boston, MA 02114, USA; 5 Gerontology Institute, Georgia State University, Atlanta, GA 30303, USA; 6 Department of Kinesiology & Health, Georgia State University, Atlanta, GA 30303, USA; 7 Nell Hodgson Woodruff School of Nursing, Emory University, Atlanta, GA 30322, USA; 8 School of Electrical and Computer Engineering, Georgia Institute of Technology, Atlanta, GA 30332, USA; 9 Department of Neurology, Emory University, Atlanta, GA 30322, USA

**Keywords:** dynamic functional connectivity (dFNC), independent component analysis (ICA), physical activity

## Abstract

**Background/Objectives:** Young adulthood is a critical developmental period during which lifestyle behaviors may shape intrinsic brain network dynamics that support cognition. This pilot longitudinal intervention study examined whether variability in physical activity and sedentary behavior during an 8-week exercise and/or cognitive intervention protocol was associated with changes in intrinsic brain dynamics and cognitive and mood outcomes in undergraduate young adults. **Methods:** Participants (n = 32) completed resting-state functional magnetic resonance imaging (rs-fMRI) at baseline (T1) and post-intervention (T2). Dynamic functional network connectivity (dFNC) was estimated from 53 intrinsic connectivity networks derived using spatially constrained independent component analysis (ICA). Ten recurring dynamic connectivity states were identified and individualized using constrained dynamic double functional independent primitives (c-ddFIPs). State occupancy and dynamic convergence and divergence metrics were computed to characterize network flexibility. **Results:** Greater moderate-to-vigorous physical activity was modestly but consistently associated with increased occupancy of integrative higher-order states, particularly States 6 and 7, and reduced occupancy of more segregated configurations. More physically active individuals also demonstrated greater divergence between integrative and low-engagement states, whereas greater sedentary time corresponded to increased similarity among segregated configurations. Working memory performance showed parallel associations with more integrative and better-differentiated dynamic patterns. **Conclusions:** These findings suggest that dynamic functional network reconfiguration may represent a neurobiological mechanism linking lifestyle behaviors and cognitive health in young adulthood. Furthermore, they highlight the translational promise of engagement-driven, low-burden programs for college-aged young adults, showing that even modest variability in habitual physical activity corresponds to greater engagement and differentiation of integrative connectivity states linked to executive and broader cognitive functions.

## 1. Introduction

The human brain exhibits ongoing fluctuations in the temporal synchrony of activity across distributed regions, supporting cognition, perception, and action. In young adults, these time-varying patterns of functional coupling—often referred to as dynamic functional connectivity—are thought to underpin neural flexibility, enabling adaptive responses to environmental demands, working memory maintenance, and decision-making [1,2,3]. This developmental period is also characterized by heightened neuroplasticity and continued maturation of higher-order cognitive systems [4], making young adulthood a critical window for identifying modifiable lifestyle factors that may support cognitive health.

A growing body of work suggests that lifestyle interventions, particularly physical exercise, can modulate intrinsic functional connectivity and relate to improved cognitive outcomes in healthy young adults [5,6,7,8]. Exercise has been associated with altered coupling within and between large-scale networks supporting attention and executive function [9,10,11], potentially through physiological pathways including increased cerebral blood flow [12] and neurotrophic mechanisms [13,14]. At the same time, many college-aged adults report high stress, anxiety, and sedentary behavior, each of which may relate to less optimal intrinsic network organization and poorer cognitive functioning, underscoring the need for scalable interventions and neurobiological metrics sensitive to lifestyle variability.

Dynamic functional network connectivity (dFNC) provides a framework for quantifying time-varying interactions among intrinsic connectivity networks during resting-state fMRI, capturing transient configurations that are not reflected in static connectivity estimates [3]. Prior work indicates that higher physical activity is associated with greater network reconfiguration and flexibility [15]. In addition, the proportion of time spent in specific dynamic connectivity states (state occupancy) has been linked to cognitive performance, with more time in integrative states relating to better attention and working memory [16] and excessive occupancy of more segregated configurations relating to poorer cognition [17]. Beyond occupancy, divergence metrics that quantify how distinctly states are expressed relative to one another have also been associated with cognitive functions such as attention and working memory [18,19], often with focused effects in particular state-pair relationships reflecting neural adaptability [20]. However, relatively few studies in young adults have combined objective physical activity monitoring with individualized dynamic connectivity modeling to characterize how exercise engagement and sedentary behavior relate to both the temporal prevalence of states and the differentiation among them.

To address this gap, we applied constrained dynamic double functional independent primitives (c-ddFIPs), an individualized dynamic connectivity framework that reconstructs subject-specific dynamic states from group-level priors and enables quantification of state occupancy as well as dynamic convergence and divergence patterns [19,21,22]. This approach is well-suited for intervention and lifestyle research in young adults because it maintains a common state space across participants while increasing sensitivity to subtle, person-specific variation in state expression.

The present study examined longitudinal resting-state fMRI data collected at baseline (T1) and post-intervention (T2) in undergraduate young adults participating in an 8-week exercise and/or cognitive intervention feasibility trial, alongside objective Fitbit-derived activity measures and NIH Toolbox cognitive outcomes. Our objectives were to: (1) determine whether individual differences in physical activity intensity and sedentary behavior across the intervention period were associated with post-intervention dFNC state occupancy, convergence, and divergence; and (2) test whether these dynamic connectivity metrics were associated with cognitive performance (Dimensional Change Card Sort, Flanker, and List Sorting Working Memory) in the same post-intervention window.

We hypothesized that greater engagement in moderate-to-vigorous physical activity would be associated with increased occupancy of adaptive/integrative connectivity states and greater divergence between integrative states and lower-engagement or more segregated configurations. Conversely, we hypothesized that greater sedentary behavior would be associated with increased occupancy of segregated or sensory-weighted states and greater convergence among lower-integration configurations. Finally, we expected that better cognitive performance—particularly working memory and executive control—would align with greater engagement and differentiation of integrative dynamic states.

## 2. Materials and Methods

### 2.1. Participants and Behavioral Measures

Participants were drawn from the Healthy Student Brain (HSB) project, a longitudinal intervention study at Georgia State University examining how aerobic exercise and online cognitive intervention influence neural dynamics, cognition, and well-being in sedentary undergraduate students. The full sample consisted of 103 students (ages 17–33), and a neuroimaging subsample of 32 individuals (ages 18–33) completed MRI scanning at baseline and following the 8-week intervention. All participants provided informed consent, and study procedures were approved by the Georgia State University Institutional Review Board. This study was designed as a pilot intervention to examine the feasibility, adherence, and preliminary effect sizes of an exercise and cognitive intervention protocol in a young adult university sample. Accordingly, the primary objective was to evaluate intervention implementation and signal detection rather than to test sex-specific biological effects. The majority of enrolled participants were female, reflecting the demographic composition of the undergraduate volunteer pool from which the sample was recruited. Recruitment occurred within a single academic setting over a defined enrollment window, and despite targeted outreach efforts, equal representation of males and females was not achieved.

### 2.2. Exercise and Cognitive Intervention

Participants were assigned to one of four intervention groups (exercise only, online cognitive intervention only, combined exercise + cognitive intervention, or control) using a pseudo-random allocation procedure designed to balance key demographic characteristics across study arms. Specifically, participants were first stratified by race and age category prior to assignment. Within each stratum, allocation was conducted sequentially to maintain proportional representation of these variables across the four groups. This approach was selected to promote demographic balance in this pilot feasibility study while preserving the practical feasibility of enrollment and scheduling. To maximize the observed effect of the interventions, only the exercise + online cognitive intervention group and controls underwent an MRI scan. In the exercise group, participants were asked to perform 30 min of moderate to vigorous physical activity at least three times per week. Participants were not restricted to particular exercises but instead were informed about types of exercises that increase heart rate, heart rate zones and ways to gauge moderate and vigorous activity based on perceived breathlessness and measures collected by the FitBit. In the brain health training condition, participants underwent brain health training created by the UT Dallas Center for BrainHealth [23]. The program consists of short, self-paced digital modules that teach evidence-informed cognitive strategies aimed at strengthening attention, reasoning, mental flexibility, and overall brain wellness. No data from this training were analyzed in the present study, only the NIH toolbox cognitive measure taken pre-and post intervention. For the purposes of this analysis, we were interested in minutes of exercise and sedentary behavior across the study and we collapsed across intervention groups. Because the present study was designed as a pilot feasibility trial rather than a fully powered randomized controlled trial, participants were not formally offered a structured post-study intervention as part of the protocol.

#### Measures

Participants were continuously monitored using a Fitbit Inspire 2 device worn throughout the 8-week study, yielding objective estimates of daily moderate-to-vigorous physical activity, sleep, and heart rate. Data were collected and managed using Fitabase [24]. Physical activity was measured continuously using Fitbit wearable devices, which classify each minute of movement into standardized intensity categories using triaxial accelerometer data and validated proprietary algorithms. The exercise intervention was designed to increase sustained moderate-to-vigorous aerobic activity in young adults over an 8-week period to examine whether such increases would be associated with measurable improvements in brain health. Brain health outcomes were operationalized as changes in cognitive performance and mood measures assessed pre- and post-intervention, including NIH Toolbox executive function tasks and validated self-report measures of stress, depression, and anxiety. The intervention was therefore structured to promote consistent cardiovascular engagement rather than short-term or sporadic activity, aligning the behavioral prescription with the hypothesized neurocognitive mechanisms of exercise-induced plasticity. For each participant, we extracted daily totals of Sedentary, Lightly Active, Fairly Active, and Very Active Minutes. Sedentary Minutes represent minimal movement consistent with sitting or reclining. Lightly Active Minutes reflect low-intensity movement (e.g., slow walking or routine daily tasks). Fairly Active Minutes correspond to moderate-intensity activity (approximately 3–6 METs; e.g., brisk walking), and Very Active Minutes represent vigorous-intensity movement (>6 METs; e.g., running or high-effort exercise). When heart rate was available, Fitbit integrates optical heart-rate data to refine the activity-intensity classification. These intensity minutes are widely used in physical activity research and have demonstrated acceptable validity for estimating free-living activity in young adults. We summed the fairly + very active minutes, as both categories represented acceptable heart rate zones that we defined as exercise in our instructions to participants. We summed across each participant for the duration of this study and used these outcome measures (total sedentary minutes, and fairly+very active minutes) in our statistical analyses. Initial descriptive analysis of our cohort indicated that controls did in fact exercise even when they were instructed not to. When investigating how many weeks participants met our recommended three times per week of moderate to vigorous exercise, all controls exceeded this recommendation for more than 1 week during the intervention period. Thus, as previously mentioned, we collapsed across all intervention groups to investigate a dose-dependent relationship between sedentary behavior and connectivity metrics, and physical activity and connectivity metrics.

Behavioral assessments were collected both online and in person pre and post intervention. Participants completed validated self-report measures of stress, depression, and anxiety, including the Perceived Stress Scale (PSS) [25] and Patient-reported outcomes measurement information system (PROMIS) emotional health domains [26]. Cognitive performance was assessed using the NIH Toolbox Dimensional Change Card Sort, Flanker, and List Sorting Working Memory tasks, providing measures of cognitive flexibility, inhibitory control, and working memory capacity, respectively.

### 2.3. MRI Data Acquisition and Preprocessing

Resting-state functional magnetic resonance imaging (fMRI) data were collected at the Georgia State/Georgia Tech Center for Advanced Brain Imaging (CABI) and the GSU Urban Life Building during baseline and post-intervention visits. Each scanning session included a high-resolution anatomical scan and a 4D resting-state fMRI acquisition. Standard MRI safety procedures were followed, and participants were included in scanning only if they met all MRI eligibility criteria.

The preprocessing pipeline included motion correction, spatial normalization, artifact reduction, and template-guided ICN estimation to maximize cross-subject comparability. The resulting ICN time courses were used to compute both static and dynamic functional network connectivity metrics, forming the basis for subsequent analyses of time-varying network organization.

### 2.4. Extraction of Intrinsic Connectivity Networks (ICNs)

To characterize large-scale functional brain networks consistently across participants, we employed a spatially constrained independent component analysis (ICA) approach guided by the Neuromark_fMRI_1.0 template [27]. Spatially constrained ICA extends traditional ICA by incorporating robust spatial priors that reduce component-order ambiguity and enhance cross-subject correspondence. This approach embeds data-driven decomposition within a framework of independently derived network templates, enabling the extraction of individualized intrinsic connectivity networks (ICNs) while retaining stability and comparability across participants.

All analyses were implemented using the GIFT toolbox at http://trendscenter.org/software/gift (accessed on 14 August 2025). For each resting-state fMRI scan, 53 ICNs were reconstructed based on NeuroMark’s standardized atlas, which has been validated for detecting reproducible functional networks across diverse cohorts and clinical populations. These networks were grouped into seven functional domains—subcortical (SC), auditory (AUD), visual (VS), sensorimotor (SM), cognitive control (CC), default mode (DM), and cerebellar networks (CB)—providing broad coverage of large-scale functional architecture.

### 2.5. Dynamic Functional Network Connectivity Estimation

Temporal variations in connectivity were quantified using a dFNC framework. For each participant and session (baseline and follow-up), the 53 ICN time courses were segmented using a 45 TR sliding window (rectangular window, 1 TR step size). Pearson correlation coefficients were calculated for each window, producing a sequence of 53×53 connectivity matrices that capture ongoing fluctuations in inter-network coupling.

To focus on dynamic fluctuations rather than static baseline connectivity, each windowed FNC matrix was demeaned. This normalization step removes the static global connectivity pattern, thereby enhancing sensitivity to transient shifts in network organization. The participant-specific dFNC matrices from all individuals and visits were concatenated to form a single group-level dataset representing population-wide temporal variability.

### 2.6. Identification of Group-Level Dynamic States (ddFIPs)

**Figure 1:**
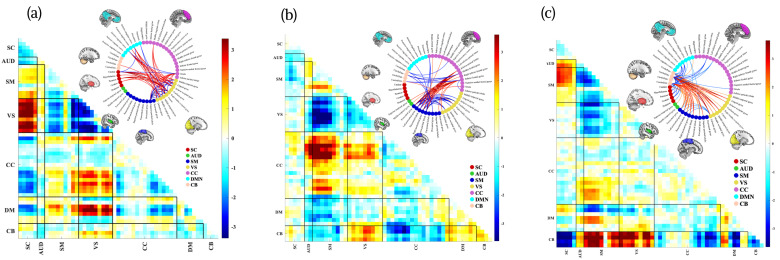
A subset of sample dynamic double functional independent primitives (ddFIPs), including (**a**) ddFIP 3, (**b**) ddFIP 7, and (**c**) ddFIP 8, along with their associated connectograms, demonstrating the variability and shared structure of dynamic functional interaction patterns across canonical brain networks. Warmer colors indicate stronger positive coupling, whereas cooler colors indicate weaker or negative coupling. Rows and columns correspond to predefined brain networks: subcortical (SC), auditory (AUD), sensorimotor (SM), visual (VS), cognitive control (CC), default mode (DM), and cerebellar (CB).

The aggregated dFNC dataset was decomposed using blind ICA to derive a set of group-level dynamic states, referred to as dynamic double functional independent primitives (ddFIPs). This analysis yielded 10 partially overlapping connectivity states, each reflecting a recurring pattern of network interactions commonly expressed across individuals presented in Supplementary Figure S1. These ddFIPs serve as functional connectivity “templates” that summarize population-level dynamic configurations and form the basis for individualized state reconstruction. For reference, a representative subsample of ddFIPs is shown in Figure 1.

These states encapsulate distinct modes of large-scale network integration and segregation and have previously been linked to cognitive performance and clinical characteristics in both healthy and psychiatric populations.

### 2.7. Subject-Level Reconstruction of Constrained dFNC States (c-ddFIPs)

To obtain individualized estimates of dynamic connectivity patterns, the 10 ddFIPs were used as spatial priors in a constrained ICA back-reconstruction procedure. This approach enforces alignment between individual-level connectivity states and the group-derived templates while preserving subject-specific variability in amplitude and expression.

For each participant, the constrained decomposition yielded time-resolved state contributions—referred to as constrained dynamic double functional independent primitives (c-ddFIPs)—which provide individualized fingerprints of dynamic connectivity. This strategy enhances interpretability by ensuring that the connectivity patterns for each participant map onto a shared set of neurobiologically meaningful states.

A final calibration step was applied to each participant’s reconstructed dFNC time series. Specifically, individual connectivity patterns were regressed onto predictors formed by the outer products of participant-specific state-mixing profiles, producing beta-weighted, variance-scaled time courses. These calibrated trajectories represent standardized measures of state engagement that are directly comparable across individuals and groups.

Following reconstruction, visit 1 profiles were regressed out from visit 2 for each participant to isolate intervention-related changes. Only participants with complete second-visit data were included in downstream analyses.

### 2.8. Quantifying Dynamic Brain Properties

We computed multiple dynamic connectivity metrics to assess how variations in cognitive performance and exercise engagement were associated with time-varying patterns of brain network activity as follows:

#### 2.8.1. State Occupancy

State occupancy quantifies the proportion of time a subject spends in a specific brain state or network configuration relative to the total scan duration. Let $X_{i}\left(t\right)$ denote the calibrated activation (amplitude) of state *i* at time window *t*, with i=1,…,M states and t=1,…,T windows. To determine which state is most influential at a given moment, we computed each state’s relative amplitude by normalizing its absolute activation against the total activity across all states:

(1) $$R_{i}\left(t\right)=\frac{|X_{i}\left(t\right)|}{\sum_{j=1}^{M}\left|X_{j}\left(t\right)\right|},\;\mathrm{for}\;\sum_{j=1}^{M}\left|X_{j}\left(t\right)\right|>0.$$

The dominant state at time *t* is then defined as:

(2) $$D\left(t\right)=\arg\max_{i\in \{1,\ldots ,M\}}R_{i}\left(t\right),$$

For each state *i*, the occupancy count is obtained by summing the number of windows in which that state is dominant:

(3) $$O_{i}=\sum_{t=1}^{T}I\left(D(t)=i\right),$$

where I(·) is the indicator meaning it equals 1 if its argument is true and 0 otherwise. The corresponding occupancy percentage is given by:

(4) $$P_{i}=\frac{O_{i}}{T}\times 100.$$

This formulation yields a distribution describing how frequently each dynamic state emerges as the leading contributor to ongoing connectivity patterns, providing a summary of the temporal prevalence and stability of specific modes of brain network coordination.

#### 2.8.2. Dynamic Convergence and Divergence

To further characterize temporal fluctuations in state dominance, we developed a dynamic convergence/divergence analysis that examines how similarly or differently states contribute to connectivity at each time window.

For each participant, let

$$X\in R^{T\times M}$$

denote the calibrated mixing matrix, where *T* is the number of windows and *M* is the number of dynamic states. The entry $X_{i}\left(t\right)$ represents the activation amplitude of state i∈{1,…,M} at window t∈{1,…,T}.

At each window *t*, the state contribution vector is defined as

(5) $$X\left(t\right)={\left(X_{1}\left(t\right),\ldots ,X_{M}\left(t\right)\right)}^{⊤}\in R^{M},$$

which captures the instantaneous prominence of all states.

To quantify similarity between states at time *t*, we computed pairwise amplitude differences

(6) $$d_{ij}\left(t\right)=\left|X_{i}\left(t\right)-X_{j}\left(t\right)\right|,\;i,j\in \left\{1,\ldots ,M\right\},\;i\neq j.$$

Small values of $d_{ij}\left(t\right)$ indicate similar state amplitudes, whereas large values reflect differential dominance. Given predefined thresholds ε>0 and θ>ε, state pairs were classified as

(7) $$\begin{array}{cc} \mathrm{Convergent} & \;\mathrm{if}\;d_{ij}(t)<\varepsilon , \end{array}$$

(8) $$\begin{array}{cc} \;\mathrm{Divergent}\; & \mathrm{if}\;d_{ij}(t)>\theta . \end{array}$$

Thresholds were selected based on empirical inspection of the distribution of $d_{ij}\left(t\right)$ across participants and windows.

This framework distinguishes moments of coordinated state engagement (convergence), where amplitudes are similar across states, from periods of heightened differentiation (divergence), where specific states dominate relative to others, thereby providing complementary measures of neural coherence and flexibility.

### 2.9. Statistical Analysis

Associations among dynamic connectivity metrics, cognitive performance, and exercise minutes were examined using general linear models (GLMs). Predictors included exercise and mood data collected before and after the intervention period, such as daily sedentary minutes (Sedentary Minutes-total), light-intensity activity (Lightly Active Minutes-total), moderate-intensity activity (Fairly Active Minutes-total), vigorous-intensity activity (Very Active Minutes-total), and combined moderate-to-vigorous activity minutes (Fairly plus-Very-total). Weekly adherence to physical activity guidelines (weeks-adherent-to-PA-guideline), defined as the number of weeks participants achieved ≥30 min of moderate or vigorous activity on at least three days (possible range: 0–8), was also included.

Cognitive predictors consisted of age-corrected NIH Toolbox scores at second visit, including Flanker Inhibitory Control and Attention accuracy (Flanker-t2-AC), List Sorting Working Memory accuracy (LSWM-T2-AC), and Dimensional Change Card Sort accuracy (DCS-T2-AC), capturing executive control, working memory capacity, and cognitive flexibility, respectively.

To control for pre-intervention cognition, stress, and connectivity, we regressed time 1 stress, cognition, and connectivity from time 2 (post intervention) data. Outcome variables consisted of state occupancy measures, convergence/divergence metrics, and calibrated state amplitudes. Models were adjusted for gender and motion where appropriate, and significance thresholds were corrected for multiple comparisons when necessary.

## 3. Results

### 3.1. Participant Characteristics

Descriptive characteristics of the full sample (N = 103) and the neuroimaging subsample n = 32) are presented in Table 1. The sample was predominantly female and racially diverse, with a mean age of approximately 21 years. There were no significant differences in age or sex distribution between the full sample and MRI subsample.

### 3.2. Dynamic State Expression Across Participants

**Figure 2:**
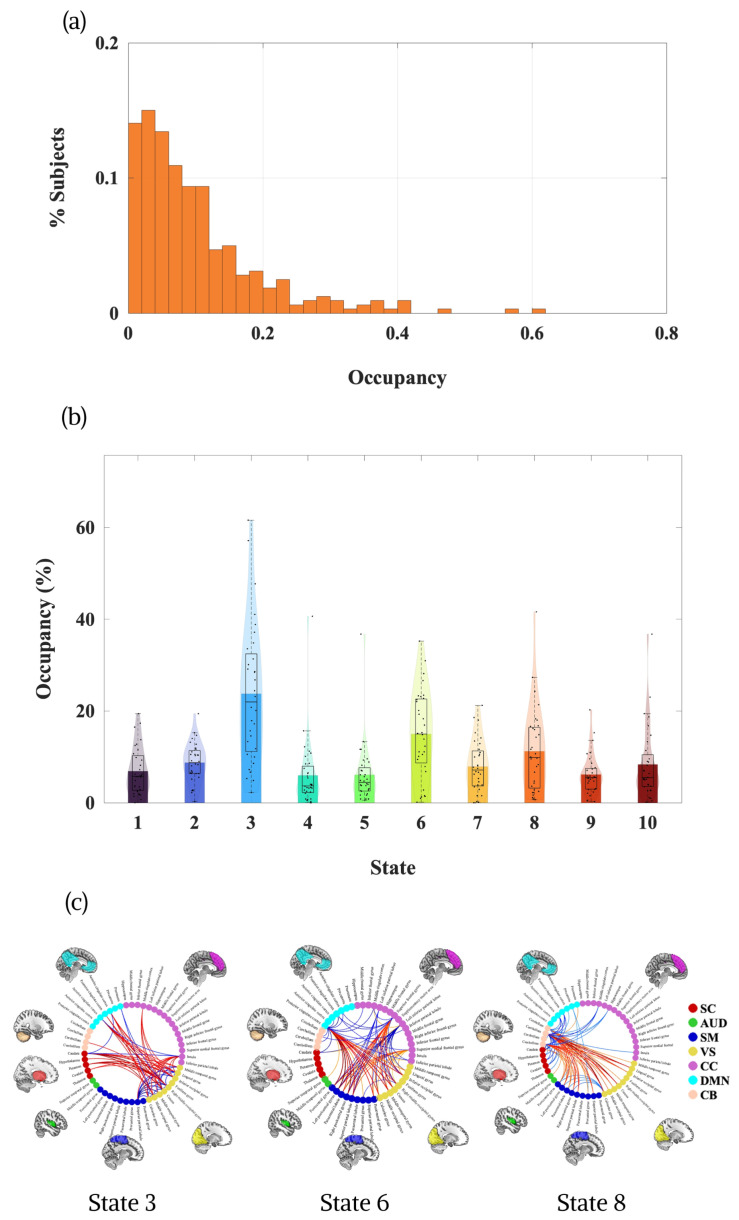
State occupancy distributions and representative dynamic connectivity configurations. (**a**) Pooled histogram of fractional occupancy across all participants and states. The distribution is right-skewed, with the majority of state expressions occurring within the 0–10% range and relatively few instances of sustained dominance. (**b**) Fractional occupancy (%) for each of the ten ddFIP-derived states across participants. Bars represent mean occupancy, boxplots indicate median and interquartile range, and violin overlays depict the full distribution. State 3 exhibited the highest occupancy and greatest inter-individual variability. (**c**) Representative connectograms of selected dynamic states (States 3, 6, and 8).

Across participants, state occupancy demonstrated a markedly right-skewed distribution, with the majority of time windows characterized by low fractional occupancy values and relatively few instances of sustained state dominance (Figure 2a). The pooled occupancy histogram indicated that most state expressions fell within the 0–10% range, with progressively fewer participants exhibiting extended durations of dominance. Only a small subset of states reached substantially higher occupancy levels, underscoring that dominant dynamic configurations were generally transient rather than persistent. This overall distribution reflects a dynamic regime marked by frequent transitions and flexible reconfiguration of large-scale networks, rather than prolonged stabilization within a single connectivity pattern.

When examined at the level of individual states (Figure 2b), pronounced heterogeneity emerged across the ten ddFIP-derived configurations. State 3 exhibited the highest mean occupancy and the greatest inter-individual variability, with several participants showing prolonged engagement exceeding 40–60%. Functionally, this state was characterized by reduced within-visual network interactions alongside enhanced coupling between the VS and SC networks. This shift suggests a reweighting of sensory processing toward subcortical integration, potentially reflecting heightened coordination between visual cortices and subcortical regulatory or salience-related structures.

States 6 and 8 demonstrated intermediate levels of occupancy with moderate dispersion, indicating that they represent recurrent but not primary configurations of resting-state dynamics. State 6 was marked by increased functional segregation between sensory systems, particularly between the SM and VS domains. This pattern reflects a more modular network organization in which sensory subsystems operate with greater independence, consistent with reduced cross-modal integration. In contrast, State 8 exhibited reduced intrinsic cerebellar (CB) correlations coupled with increased connectivity between CB–SM and CB–VS (Figure 2c). This configuration suggests a redistribution of cerebellar engagement from internally cohesive processing toward enhanced cross-domain coordination with cortical sensory systems.

### 3.3. Associations Between Dynamic State Occupancy and Physical Activity

**Figure 3:**
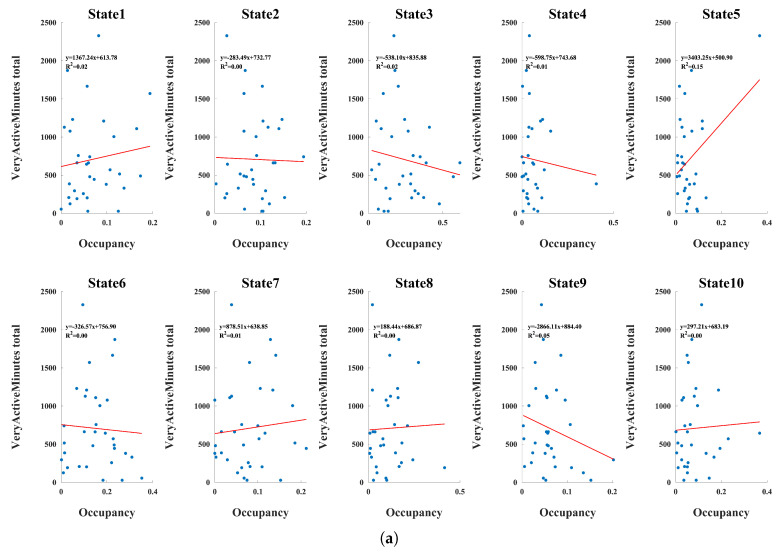

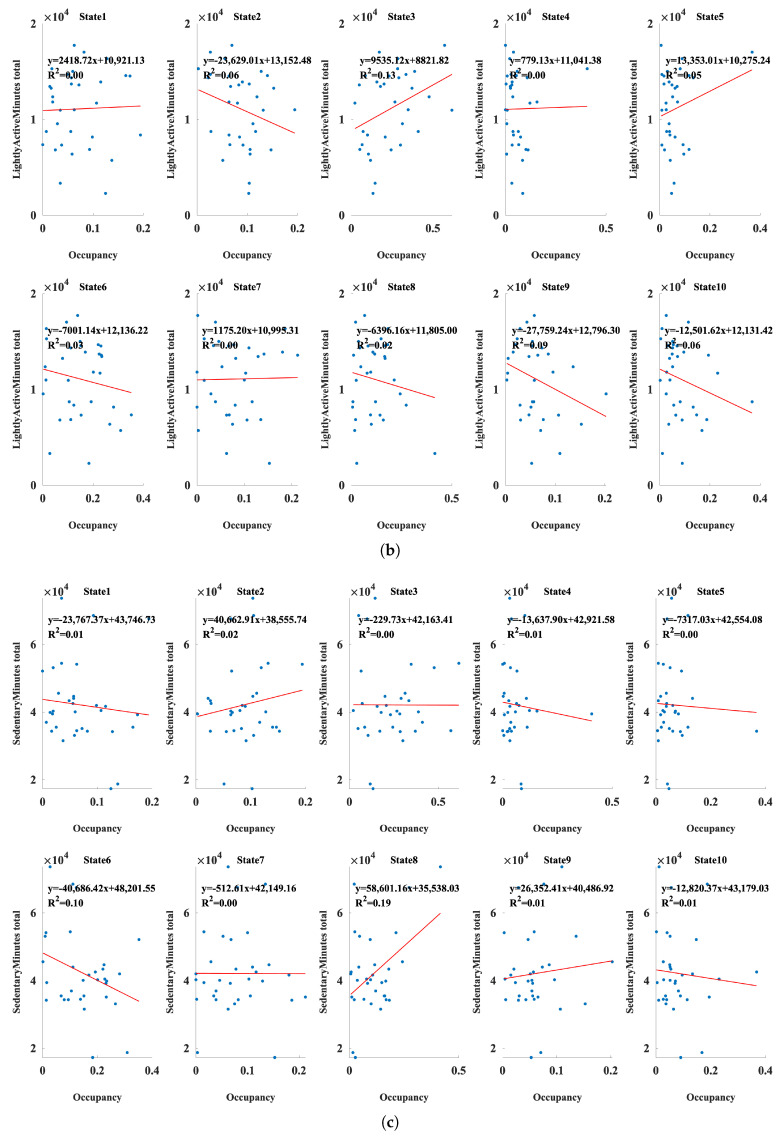
Associations between dynamic state occupancy and physical activity. Each panel depicts the relationship between occupancy of the ten dynamic connectivity states and physical activity, with individual data points shown across participants and linear fits overlaid. (**a**) Very active minutes; (**b**) Lightly active minutes; (**c**) Sedentary minutes; Across metrics, effect sizes were small, though several trends indicate that higher physical activity and better adherence were associated with greater occupancy of integrative states and reduced occupancy of segregated or sensory-weighted states.

Associations between state occupancy and objective physical activity metrics collected across the intervention period is examined using GLM (Figure 3). Across outcomes, effect sizes were modest and variability was substantial, consistent with the relatively small sample size. Nevertheless, several directional trends were observed that were coherent across related activity measures.

Higher levels of moderate-to-vigorous physical activity, indexed by very active minutes and the combined fairly + very active minutes, were associated with increased occupancy of specific dynamic states and reduced occupancy of others. In particular, states previously characterized by greater cross-network integration showed positive associations with moderate-to-vigorous physical activity, whereas states reflecting greater sensory segregation or domain-specific weighting exhibited inverse trends. Although individual slopes were small, the consistency of directionality across panels suggests that habitual engagement in higher-intensity physical activity may subtly bias resting-state dynamics toward more integrative large-scale configurations.

Intensity-specific analyses further indicated a graded pattern. Associations were more pronounced for very active minutes relative to fairly active or lightly active minutes, suggesting that higher physiological load may exert stronger influences on dynamic network organization. In contrast, sedentary time demonstrated opposing trends, with greater sedentary behavior associated with increased occupancy of more segregated or sensory-dominant states and reduced occupancy of integrative configurations. This reciprocal pattern supports the interpretation that physical activity and inactivity may shift the balance between integration and segregation within the dynamic functional connectome.

In the present data, states exhibiting increased cerebellar–sensorimotor and cerebellar–visual coupling showed positive associations with higher-intensity activity, suggesting enhanced motor–sensory integration during rest. Conversely, states characterized by greater sensory segregation or reduced cross-network coupling were more prevalent in individuals with higher sedentary exposure, potentially reflecting reduced dynamic flexibility. Figures corresponding to all GLM results are available in the Supplementary Materials.

### 3.4. Associations Between Dynamic State Occupancy and Cognitive Performance

**Figure 4:**
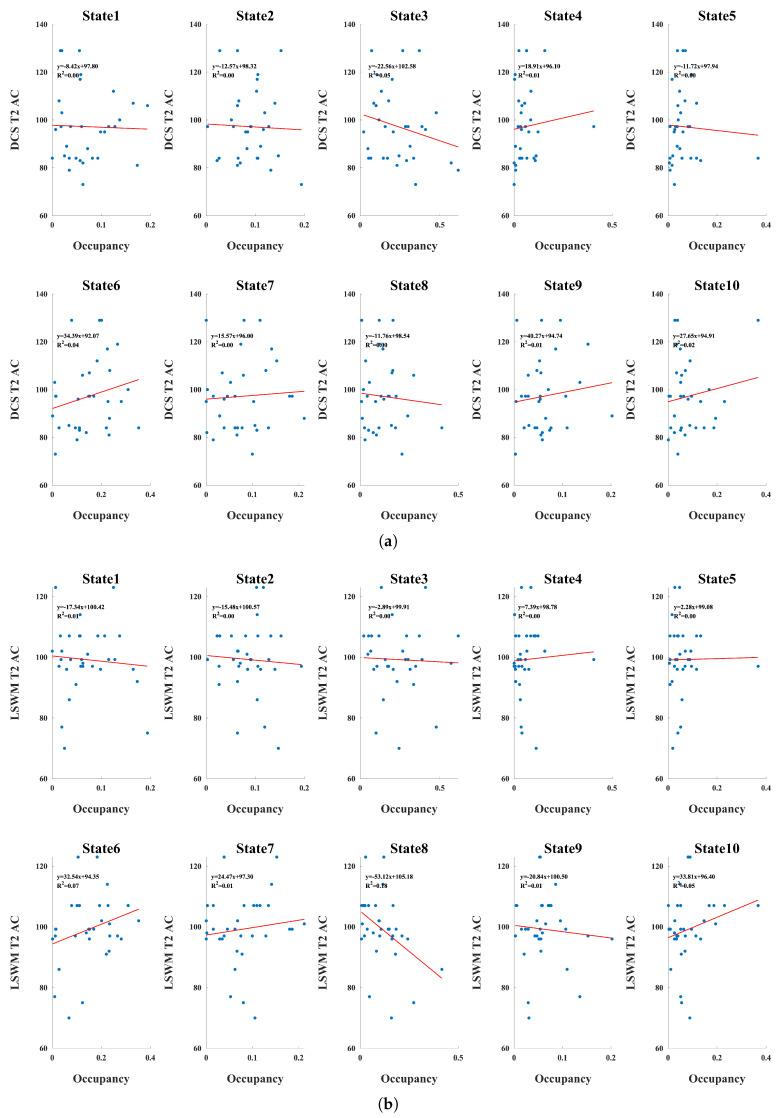

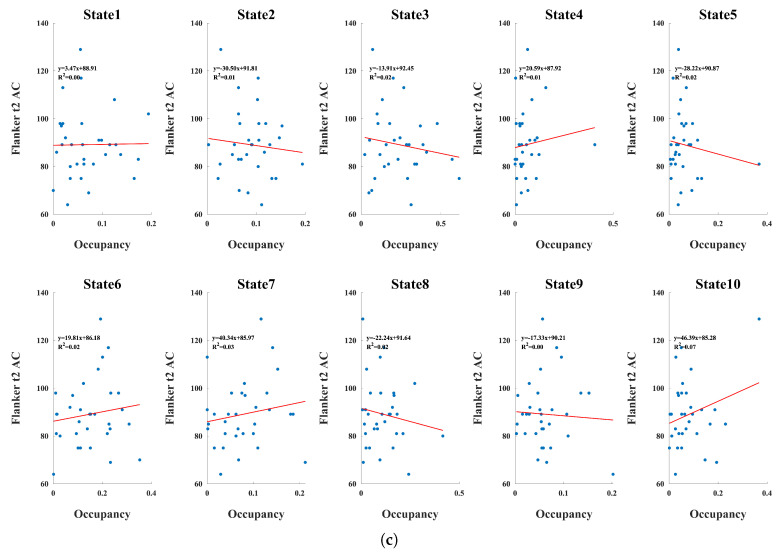
Associations between dynamic state occupancy and cognitive performance outcomes. Each panel shows linear associations between occupancy of the ten dynamic connectivity states and a cognitive metric, with individual participants represented as points and regression lines overlaid. (**a**) Age-corrected dimensional change card sort accuracy score at Time 2; (**b**) List Sorting Working Memory (LSWM) accuracy at Time 2 (**c**) Flanker inhibitory control and attention accuracy at Time 2.

State-specific associations between dynamic occupancy and cognitive outcomes revealed dissociable patterns across executive domains (Figure 4). State 3 demonstrated a consistent negative association with both DCS and Flanker performance, while showing a comparatively flat relationship with LSWM. Given that State 3 is characterized by increased VS-SC coupling and reduced within-visual coherence, greater occupancy of this configuration may reflect a redistribution of sensory processing toward subcortical integration at the expense of locally specialized visual network stability. Such a configuration may be less optimal for tasks requiring rapid rule switching (e.g., DCS) and conflict monitoring (e.g., Flanker), which depend on efficient coordination between higher-order control systems and stable sensory representations, thereby accounting for the observed negative slopes. The absence of a clear association with LSWM suggests that this sensory–subcortical integration pattern may be less directly involved in sustained working memory maintenance.

In contrast, State 9 exhibited decreasing associations for both DCS and LSWM but an opposing (positive) association for Flanker performance. Enhanced CC–VS interaction in state 9 may support top-down modulation of perceptual representations, potentially benefiting attentional control processes engaged during the Flanker task. However, reduced SC–CC integration may limit flexible updating or subcortical gating mechanisms that contribute to cognitive flexibility and working memory, consistent with the negative associations observed for DCS and LSWM.

State 8 showed a comparatively steep negative association with LSWM performance. The cerebellum has been implicated in predictive coordination and working memory processes through its modulatory influence on distributed cortical networks. Reduced CB–SC integration within this configuration may reflect diminished cerebellar contributions to subcortical gating or updating mechanisms necessary for working memory maintenance, potentially explaining the pronounced negative slope observed for LSWM.

Taken together, these findings indicate that the cognitive relevance of dynamic states depends on their underlying network architecture. Rather than uniformly predicting better or worse outcomes, increased time spent in specific dynamic regimes appears to differentially influence cognitive flexibility, inhibitory control, and working memory as a function of large-scale integration and segregation patterns.

### 3.5. Threshold-Free Distance Distribution

**Figure 5:**
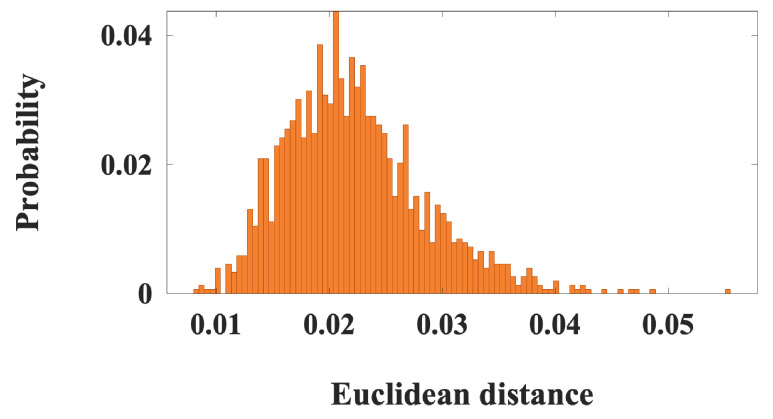
Distribution of pairwise Euclidean distances between state amplitudes across all subjects and time windows. The unimodal, right-skewed distribution indicates that most state pairs differ only modestly in amplitude at any given moment, with larger separations occurring less frequently. This empirical distribution was used to guide the selection of convergence and divergence thresholds, with lower-bound thresholds capturing genuinely close similarity and upper-bound thresholds capturing only the most pronounced state separations.

Prior to defining convergence and divergence, we examined the empirical distribution of all pairwise Euclidean distances between state amplitudes across subjects and time windows. The resulting histogram shown in Figure 5 exhibited a unimodal, right-skewed profile, with the majority of distances falling within a relatively narrow range around a small value (on the order of 0.02) and a gradual tail extending toward larger distances. This pattern suggests that, at any given moment, most state pairs differ only modestly in amplitude and that very large separations between states are comparatively rare.

On the basis of this observed pattern, the convergence (ε) and divergence (θ) thresholds were chosen with reference to the empirical distribution: ε was placed near the lower shoulder of the density to capture genuinely tight similarity, and θ was placed in the upper tail to identify only the most pronounced separations. Thus, convergent and divergent events reflect meaningful deviations from typical state differences rather than arbitrary cutoffs.

### 3.6. Convergence-Based Dynamic Metrics

We next examined convergence, quantifying how often pairs of states expressed similar amplitudes over time. The mean convergence matrix indicated that convergence was not uniform across the dynamic repertoire: a cluster of mid-range states (States 4–7) showed relatively higher mutual convergence than early (States 1–2) or late segregated states (e.g., State 10). This pattern is consistent with the interpretation of States 4–7 as partially overlapping, integrative configurations that frequently co-fluctuate, whereas more modular states tend to remain dynamically distinct.

**Figure 6:**
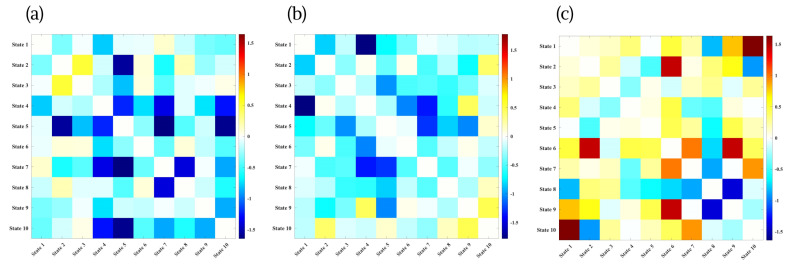
Convergence-based associations between physical activity measures and dynamic functional connectivity states, quantified using $-\log_{10}\left(p\right)\times \mathrm{sign}\left(t\right)$ for (**a**) Very active minutes; (**b**) Lightly active minutes; (**c**) Sedentary minutes. Each panel displays the statistical strength and direction of associations between physical activity and pairwise state convergence, reflecting how often pairs of dynamic states exhibit similar amplitudes over time. Higher sedentary behavior was associated with increased convergence among lower-integration states, whereas greater physical activity tended to correspond to reduced convergence between segregated and integrative states, indicating enhanced differentiation of higher-order network dynamics in more active individuals.

Behaviorally, convergence effects were generally modest but revealed a structure that was not apparent from occupancy alone. For physical activity as shown in Figure 6, convergence involving integrative states showed opposite patterns for active versus sedentary behavior. Participants with more sedentary lifestyles showed more convergence among lower-integration states (e.g., pairs involving States 2 and 10) and with convergence between these segregated configurations and the CC–DM state (State 6). In contrast, more active individuals tended to show reduced convergence between segregated and integrative states, suggesting greater differentiation between low-engagement and higher-order network patterns in those with less sedentary time.

**Figure 7:**
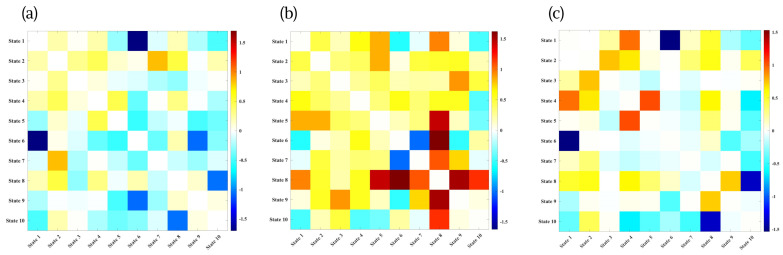
Convergence-based associations between cognitive performance and dynamic functional connectivity states, quantified using $-\log_{10}\left(p\right)\times \mathrm{sign}\left(t\right)$ for: (**a**) Age-corrected dimensional change card sort accuracy score at Time 2; (**b**) List Sorting Working Memory (LSWM) accuracy at Time 2; (**c**) Flanker inhibitory control and attention accuracy at Time 2. Each panel presents the statistical associations between cognitive outcomes and pairwise state convergence, reflecting the extent to which pairs of dynamic states exhibit similar amplitudes over time. Higher cognitive performance was generally associated with greater convergence among integrative, mid-range states, whereas lower performance tended to correspond to stronger convergence between segregated or sensory-weighted states and executive–default configurations.

Cognitive performance showed the clearest relationships with convergence (Figure 7). For working memory (LSWM), the strongest positive associations appeared for convergence among later, more integrative states (roughly within the State 5–9 cluster), whereas higher LSWM accuracy was associated with reduced convergence for specific pairs involving the CC–DM state (State 6). This pattern suggests that better working memory is linked to coordinated expression of several higher-order network configurations, while maintaining differentiation between certain executive-related states. Flanker accuracy showed weaker but directionally similar trends, with better performance associated with convergence among integrative states and reduced convergence involving State 6 and low-integration configurations.

Taken together, the convergence analysis complements the occupancy findings by indicating that it is not only the amount of time spent in a given state that matters, but also how similarly or distinctly states are expressed relative to one another. Higher physical activity and better cognitive performance are associated with a dynamic system in which integrative states co-fluctuate, while segregated states remain relatively differentiated, consistent with the notion that neural flexibility depends on both state prevalence and the degree of separation or blending between states over time.

### 3.7. Divergence-Based Dynamic Metrics

Divergence reflects the degree to which pairs of dynamic states differ in their temporal activation patterns, providing a complementary lens through which to view the distinctiveness or separation of functional configurations. Across participants, divergence matrices demonstrated clearer structure than convergence results, with several state pairs showing consistent and behaviorally meaningful differentiation.

A central observation was that State 6, characterized by strong CC–DM integration, functioned as a major hub of divergence across multiple behavioral outcomes. Divergence between State 6 and several other states (notably States 4, 5, 7, 9, and 10) was reliably associated with both physical activity and cognitive performance. These findings parallel the convergence results by indicating that individuals with higher physical activity levels or better cognitive scores maintain State 6 as a more distinct, high-integration attractor, differentiating it from more modular or sensory-weighted configurations.

Physical activity measures showed a consistent pattern in which higher levels of moderate and vigorous activity were associated with increased divergence among mid- to high-integration states, particularly those centered on States 4, 5, and 7. This suggests that more active individuals sustain greater functional separation between higher-order integrative states, in line with theories linking physical fitness to enhanced neural flexibility. Conversely, sedentary behavior was associated with reduced divergence among these states, implying diminished dynamic differentiation.

Cognitive outcomes exhibited similar trends. Working memory accuracy (LSWM) was positively related to divergence among several higher-order states, especially within the State 4–8 range, indicating that successful cognitive performance is supported not only by coordinated activity among integrative states but also by preservation of clear boundaries between these configurations and lower-integration states. Flanker accuracy showed weaker but directionally similar effects, again illustrating the role of divergence around State 6.

Collectively, the divergence findings complement occupancy and convergence metrics by demonstrating that neural flexibility is supported not only by time spent in particular states or their degree of co-fluctuation, but also by the system’s ability to maintain functional specialization across its dynamic repertoire. Greater behavioral health is therefore associated with a dynamic landscape in which high-integration states remain both well utilized and well differentiated from more modular configurations.

## 4. Discussion

This pilot study used individualized dFNC metrics to examine how objectively monitored physical activity and sedentary behavior relate to the temporal organization of intrinsic brain networks in healthy young adults. Overall, the pattern of findings suggests that higher engagement in moderate-to-vigorous physical activity corresponds to a resting-state dynamic profile characterized by greater engagement of integrative, higher-order configurations (most notably States 6 and 7) and greater differentiation between integrative versus lower-engagement or more segregated configurations. In contrast, greater sedentary time was associated with a dynamic profile marked by increased similarity among lower-integration states, consistent with a less differentiated connectivity landscape. Although effect sizes were modest, the convergence of occupancy- and divergence-based results supports the interpretation that lifestyle variability in young adulthood is reflected more in how connectivity patterns are temporally expressed and differentiated than in broad, static shifts in connectivity strength.

### 4.1. Physical Activity and Integrative Dynamic States: Interpretation in the Context of Prior Work

The observed tendency for physically active individuals to spend more time in integrative states aligns with exercise neuroscience literature, indicating that aerobic fitness is associated with more coordinated communication among large-scale networks supporting cognitive control and goal-directed behavior [28,29]. Importantly, the present results extend this literature by emphasizing a dynamic mechanism: physical activity may not simply increase overall connectivity, but may bias the brain toward spending more time in configurations characterized by greater cross-network integration. Such temporal reweighting is consistent with frameworks in which fitness supports the ability to flexibly engage distributed control-related circuitry when needed, even at rest.

Beyond occupancy, convergence/divergence patterns provided convergent evidence that physically active individuals maintained sharper separation between higher-order integrative states and more segregated or low-engagement configurations. Prior studies have similarly suggested that higher cardiorespiratory fitness relates to stronger differentiation between strongly connected, task-positive or high-engagement states and weakly connected states [5,30]. In this context, the present divergence findings suggest that one hallmark of a more adaptive dynamic repertoire may be selective integration with preserved distinctiveness: integrative configurations are engaged, but they remain well differentiated from lower-engagement configurations rather than blending into a homogenized state landscape. This distinction is conceptually important because excessive similarity among states could reflect reduced specialization or diminished dynamic range rather than improved coordination.

### 4.2. Dynamic State Properties and Cognition: Working Memory as a Convergent Behavioral Signal

The strongest behavioral convergence emerged for working memory, where better performance aligned with dynamic signatures suggestive of greater state flexibility and differentiation. This pattern is consistent with the idea that executive cognition depends on adaptive transitions between segregated processing and integrative control architectures [31]. From that perspective, spending more time in integrative states and maintaining clear differentiation among dynamic configurations may reflect a system that can more readily recruit coordinated, higher-order processing while preserving the capacity to return to specialized configurations.

The present findings also map onto prior work suggesting that dynamic network configurations observed at rest can resemble those engaged during successful cognitive performance [12,32]. While the current study did not test task-evoked state dynamics directly, the observed alignment between dynamic metrics and working memory supports the hypothesis that intrinsic dynamics can index readiness or efficiency of control-related network engagement. Mechanistically, these associations are plausible in light of evidence linking exercise to neurobiological pathways relevant to cognition, including vascular and neurotrophic support of circuitry implicated in executive processes [33,34]. Notably, the activity–dFNC relationships persisted after accounting for perceived stress, suggesting that lifestyle-related differences in dynamic organization are not solely secondary to differences in psychological distress, even though stress may still contribute to cognitive outcomes.

### 4.3. Why Effects Were Modest and Why Dynamic Metrics May Still Be Informative

A key feature of the present results is that effects were generally small and variable across individuals, which is expected in a feasibility-oriented pilot with a modest neuroimaging sample. Several factors likely contribute to the modest effect sizes. First, the intervention was designed to be pragmatic and scalable, prioritizing real-world adherence over tight control of modality and intensity; this approach enhances ecological validity but can reduce experimental contrast. Second, as noted, participants in the control condition engaged in physical activity during the study period, limiting the degree to which any between-group intervention effect could be isolated. In this context, the value of the present analyses is the emphasis on dose-related associations: even without large between-group contrasts, objectively measured behavioral variability corresponded systematically (albeit modestly) to dynamic state properties.

Importantly, the divergence and convergence metrics add interpretive depth beyond occupancy alone. Occupancy indicates how frequently a configuration is dominant, but convergence/divergence capture whether states are expressed as distinct modes versus blended patterns over time. The present findings suggest that this distinction is behaviorally meaningful: physical activity and cognition were most consistently associated with a dynamic landscape in which integrative states are both utilized and differentiated, whereas sedentary behavior corresponded to greater similarity among lower-integration configurations. This supports the broader interpretation that neural flexibility is not only about which states occur, but also about the dynamic separability of those states over time.

### 4.4. Limitations

Several limitations should be considered when interpreting these findings. First, the MRI sample size was modest, and the study was not powered to detect small effects or interaction effects (e.g., moderation by sex). As a result, the study may be underpowered for identifying subtle intervention-related changes, increasing the risk of Type II error (false negatives). In addition, effect size estimates derived from small samples may be less stable and more sensitive to individual variability, which may limit generalizability. The present findings should therefore be interpreted as preliminary and hypothesis-generating. Replication in larger, adequately powered cohorts will be necessary to confirm the robustness and reproducibility of the observed associations. Second, despite the use of objective activity monitoring, variability in moderate-to-vigorous physical activity (particularly at higher intensities) was constrained relative to sedentary time, which may limit sensitivity for intensity-specific effects. Third, adherence and behavior change were likely influenced by study participation and wearable monitoring; wearing a Fitbit may have increased awareness of activity across groups, reducing separation between intervention and control conditions. Fourth, while c-ddFIPs provide individualized state reconstruction within a shared state space, deriving 10 states from group-level priors may limit detection of highly idiosyncratic configurations. Finally, generalizability may be limited by recruitment from a single university setting and the demographic composition of the sample.

### 4.5. Future Directions

Future work should build on these preliminary findings in larger, adequately powered trials with clearer experimental contrast and longer follow-up periods. Combining objective activity monitoring with more structured manipulation of training dose (e.g., supervised sessions or tighter intensity targets) would help clarify causal pathways linking moderate-to-vigorous physical activity to changes in dynamic network organization. Multimodal neuroimaging approaches may further strengthen mechanistic inference by relating dFNC flexibility to structural connectivity and vascular or neurochemical proxies. Finally, future studies should explicitly test moderators of intervention response (including sex, baseline fitness, and genetic factors such as BDNF-related variation) and evaluate whether dynamic state metrics provide sensitive biomarkers for tracking neurocognitive change over time.

## 5. Conclusions

In this pilot feasibility study of young adults, objectively measured physical activity and sedentary behavior were associated with meaningful differences in intrinsic brain dynamics. Greater moderate-to-vigorous physical activity corresponded to increased temporal engagement of integrative connectivity states and greater differentiation between integrative and lower-engagement configurations, whereas greater sedentary time corresponded to increased similarity among lower-integration states. Working memory performance showed parallel relationships with these dynamic signatures. Together, these findings suggest that dynamic functional network reconfiguration—captured through occupancy and convergence/divergence metrics derived from c-ddFIPs—may represent a sensitive neurobiological pathway through which lifestyle behaviors relate to cognitive health during young adulthood.

## Figures and Tables

**Table 1:** Demographic Characteristics of the Full Sample and MRI Subsample.

Characteristic	Full Sample (N = 103)	MRI Subsample (n = 32)
**Age (years)**		
Mean (SD)	20.81 (3.1)	21.22 (3.2)
Range	17–33	18–33
**Gender**		
Female	77 (74.8%)	24 (75.0%)
Male	23 (22.3%)	8 (25.0%)
Nonbinary/Other	3 (2.9%)	0 (0%)
**Race/Ethnicity**		
Asian	27 (26.2%)	10 (31.3%)
Black/African American	42 (40.8%)	10 (31.3%)
White/Caucasian	14 (13.6%)	5 (15.6%)
Hispanic/Latino	9 (8.7%)	6 (18.8%)
Biracial (Black–White)	2 (1.9%)	1 (3.1%)
Other/Multiracial	9 (8.7%)	0 (0%)

## Data Availability

The data supporting the findings of this study are available from the corresponding author upon reasonable request, subject to privacy and ethical restrictions.

## Supplementary Materials

The following supporting information can be downloaded at: https://www.mdpi.com/article/10.3390/brainsci16030327/s1. Figure S1. Complete set of sample dynamic double functional independent primitives (ddFIPs), spanning ddFIP 1 through ddFIP 10 respectively (panels a–j), illustrating the full spectrum of dynamic functional interaction patterns across canonical brain networks. Figure S2. Associations between dynamic state occupancy and physical activity.
