# Supplementary material for: Activity Patterns in Relation to Dynamic Functional Network States: A Longitudinal Feasibility Study of Brain–Behavior Associations in Young Adults
Source: Brain Sci. 2026 Mar 19;16(3):327. doi: 10.3390/brainsci16030327 (PMC13024018; doi:10.3390/brainsci16030327)
Supplement: Supplementary file 1 [file brainsci-16-00327-s001.zip › brainsci-4108668-supplementary.pdf]

Supplementary Materials S1

Comprehensive Overview of Intervention Protocol and Study Measures

1. Study Overview and Intervention Objective

This study was designed as a pilot randomized feasibility intervention to evaluate whether increasing moderate-to-vigorous aerobic exercise over an 8-week period would be associated with improvements in brain health in a young adult university sample.

Brain health outcomes were operationalized as changes in cognitive performance and mood and psychological well-being measures.

2. Exercise Intervention Overview

Participants completed 3 sessions per week, 30 minutes per session, for 8 weeks (target dose: 24 sessions). Each session included a 5-minute warm-up, 30-minute continuous moderate-to-vigorous aerobic activity, and a 3–5-minute cool-down.

Intensity was verified using Fitbit-derived moderate-to-vigorous heart rate zones or Rating of Perceived Exertion (RPE 12–16).

3. Brain Health Training Intervention Overview

Participants completed 30 minutes per week of structured cognitive training for 8 weeks (target dose: 240 total minutes).

4. Behavioral and Cognitive Measures

All instruments were standardized and validated measures. Primary brain health outcomes included cognitive performance and mood measures.

5. Recruitment

Study Flyers were placed in high-traffic, visible areas around the Georgia State University Main campus and the Georgia Perimeter College Clarkston and Dunwoody campuses after approval was given from the Student Life Council Office to recruit undergraduate students who were sedentary in their activity levels. If an undergraduate student was interested in participating, they scanned a QR code on the flyer, which took them to the Goodin Leisure Time Exercise Questionnaire to complete for eligibility. We also received approval to use the GSU digital messaging boards and blast email system to recruit our undergraduate students across all campuses, including a digital copy of the flyer with the same QR code.

6. Goodin Leisure Time Exercise Questionnaire (GLTEQ)

The Goodin Leisure Time Exercise Questionnaire asked each undergraduate student who scanned the QR code during a typical 7-Day period in a week how many times, on average, they engage in strenuous, moderate, or mild-to-light exercise for more than 15 minutes during their free time. A weekly leisure activity score of 9 was used for strenuous, 5 for moderate, and 3 for mild to light for each instance the student performed each type of exercise. Then, the scores were tallied, and the Godin Scale Score Interpretation was used to evaluate each student's exercise level. The participant was classified as active (score of 24 or more), moderately active (score of 14-23), or insufficiently active or sedentary (score of less than 14). The research coordinator then placed a follow-up call to each student who scored below 14, the study's exclusion setpoint for further evaluation.

At T1, participants in the exercise group were given the attached exercise handout and a Fitbit. The instructions given by the research assistant were to wear the Fitbit 24 hrs a day, 7 days a week, for the entire 8-week period, and only take it off to shower and charge. Each participant set up their own Fitbit before leaving the session and authorized the study to monitor their activity through the Fitbit database.

7. *NIH Toolbox Cognitive Assessments Used in Primary Analyses*

The following age-corrected scores from the NIH Toolbox Cognition Battery were used in the present analyses. All scores are standardized T-scores (Mean = 50, SD = 10), with higher scores indicating better performance.

- Dimensional Change Card Sort (DCCS): Age-corrected accuracy score at Time 2.
- List Sorting Working Memory (LSWM): Accuracy score at Time 2.
- Flanker Inhibitory Control and Attention Test: Accuracy score at Time 2.

For primary analyses, age-corrected Time 2 scores were residualized against corresponding age-corrected Time 1 scores to model post-intervention performance while accounting for baseline levels.

Given the pilot nature of the study, analyses emphasized feasibility, adherence, and preliminary effect size estimation rather than definitive causal inference.

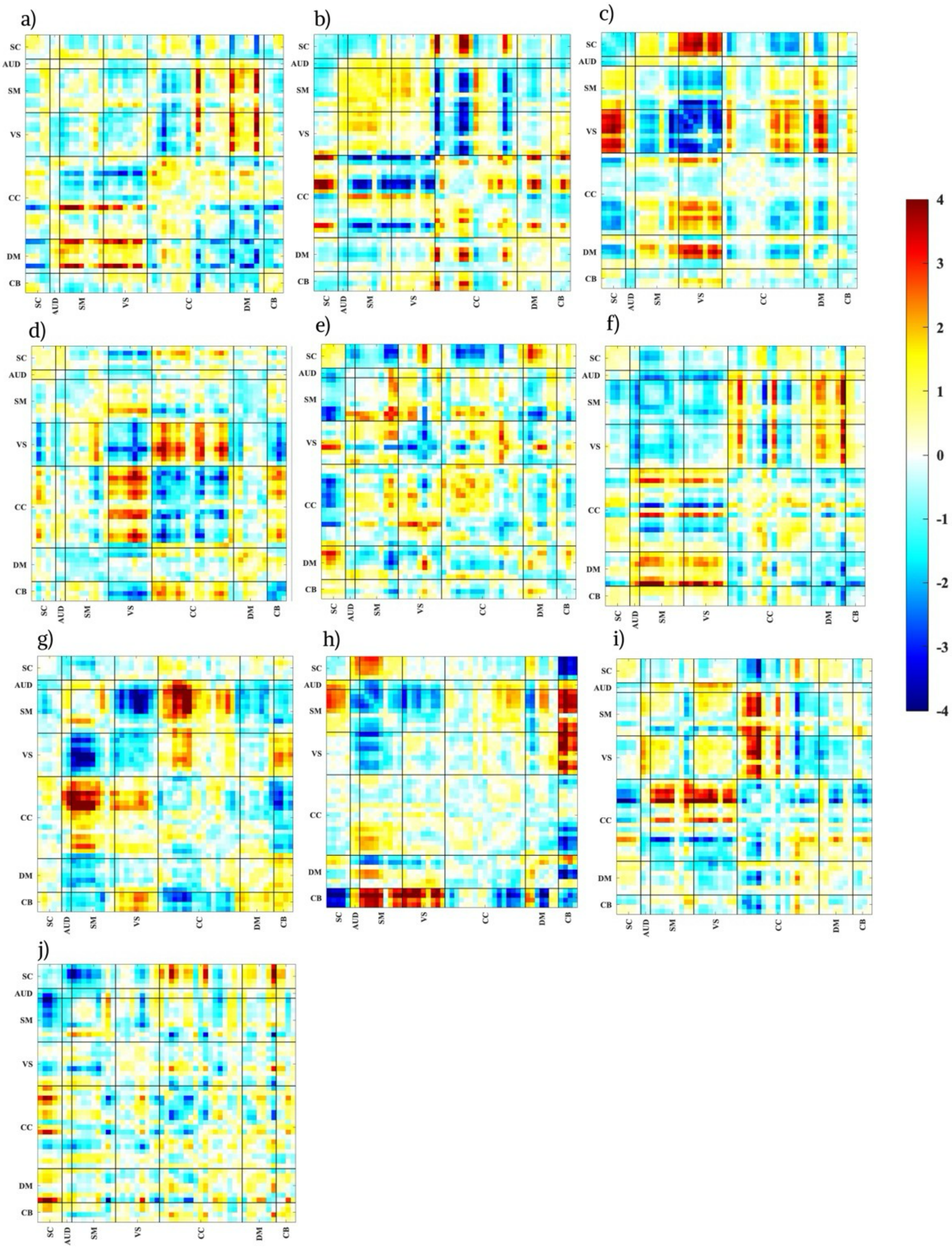

Figure S1. Complete set of sample dynamic double functional independent primitives (ddFIPs), spanning ddFIP 1 through ddFIP 10 respectively (panels a–j), illustrating the full spectrum of dynamic functional interaction patterns across canonical brain networks. Warmer colors represent stronger positive coupling, whereas cooler colors indicate weaker or negative coupling. Rows and columns correspond to predefined brain networks: subcortical (SC), auditory (AUD), sensorimotor (SM), visual (VS), cognitive control (CC), default mode (DM), and cerebellar (CB).

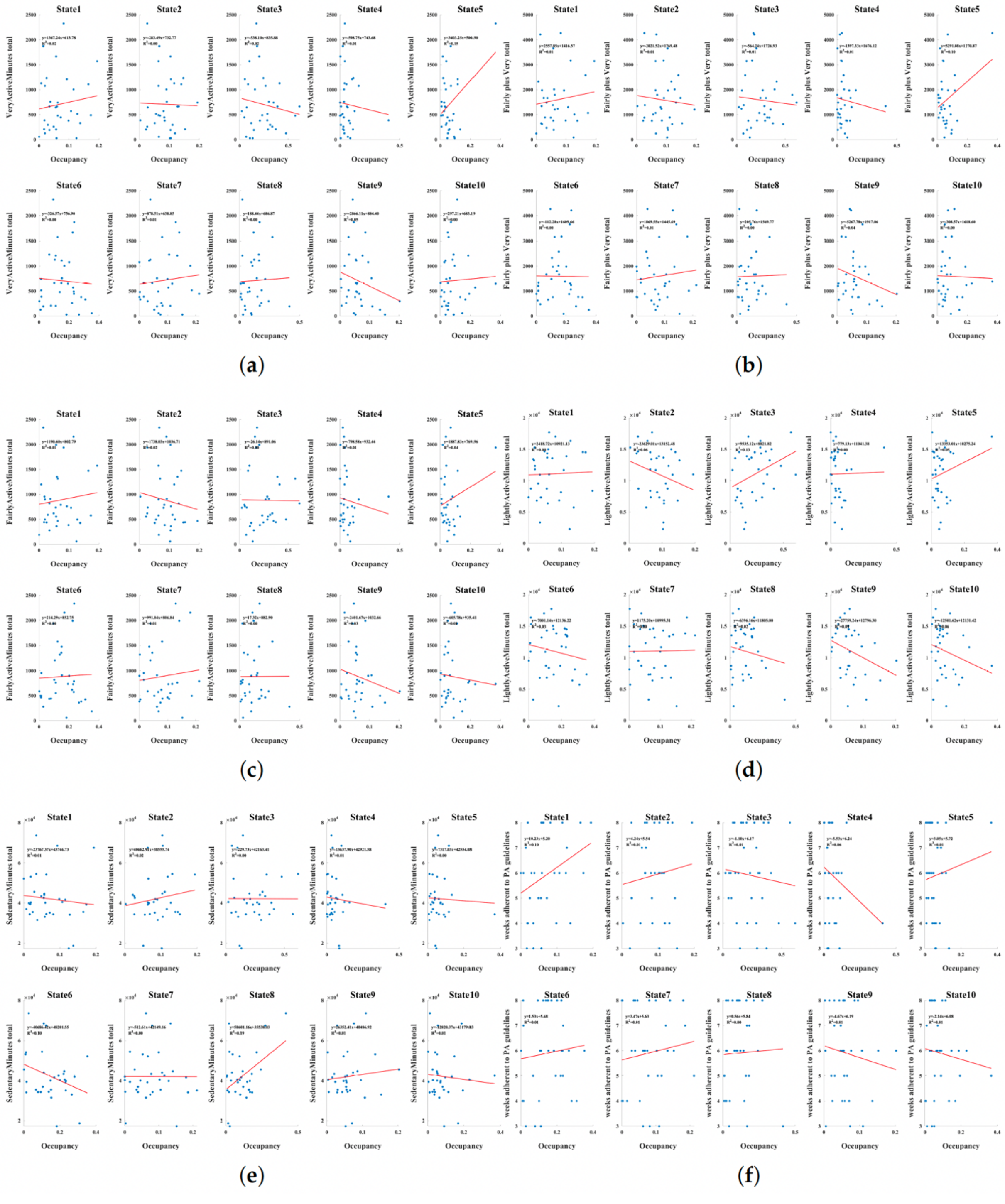

Figure S2. Associations between dynamic state occupancy and physical activity. Each panel depicts the relationship between occupancy of the ten dynamic connectivity states and a behavioral outcome, with individual data points shown across participants and linear fits overlaid. (a) Very active minutes; (b) Combined fairly active and very active minutes; (c) Fairly active minutes; (d)Lightly active minutes; (e) Sedentary minutes; (f) Number of weeks adherent to recommended physical activity guidelines. Across metrics, effect sizes were small, though several trends indicate that higher physical activity and better adherence were associated with greater occupancy of integrative states and reduced occupancy of segregated or sensory-weighted states.
